# Venom peptides – A comprehensive translational perspective in pain management

**DOI:** 10.1016/j.crtox.2021.09.001

**Published:** 2021-09-09

**Authors:** Vidya V, Raghu Ram Achar, Himathi M.U, Akshita N, Yogish Somayaji T, Vivek Hamse Kameshwar, K. Byrappa, Dinesha Ramadas

**Affiliations:** a K. S Hegde Medical Academy, NITTE (Deemed to be) University, Mangalore 575015, Karnataka, India; b Division of Biochemistry, School of Life Sciences, JSS Academy of Higher Education & Research, S.S. Nagar, Mysuru 570 015, Karnataka, India; c Department of Post Graduate Studies and Research in Biochemistry, St. Aloysius College (Autonomous), Mangalore 575003, Karnataka, India; d School of Natural Science, Adichunchanagiri University, B.G. Nagara-571448, Nangamangala, Mandya, India; e School of Natural Sciences, ACU-CRI, Adichunchanagiri University, BGSIT Campus, B.G. Nagara-571448, Nagamangala, Mandya, India; f Adichunchanagiri Institute for Molecular Medicine, AIMS, Adichunchanagiri University, B.G. Nagara-571448, Nagamangala, Mandya, India; g Center for Material Science and Technology, Vijnana Bhavan, University of Mysore, Mysuru, Karnataka, India

**Keywords:** Snake venom, Toxicity, Toxins, Inflammation, Peptides, Pain, Nociception

## Abstract

Venom peptides have been evolving complex therapeutic interventions that potently and selectively modulate a range of targets such as ion channels, receptors, and signaling pathways of physiological processes making it potential therapeutic. Several venom peptides were deduced *in vivo* for clinical development targeting pain management, diabetes, cardiovascular diseases, antimicrobial activity. Several contributions have been detailed for a clear perspective for a better understanding of venomous animals, their venom, and their pharmacological effects. Here we unravel and summarize the recent advances in wide venom peptides across varieties of species for their therapeutics prospects.

## Highlights

• A comprehensive review about number of toxins with pharmacological importances.
• Toxicological evaluations of several toxins with its targeted protein.
• Benchmark of venom peptides with mechanism of action.

## Graphical abstract

**Figure:**
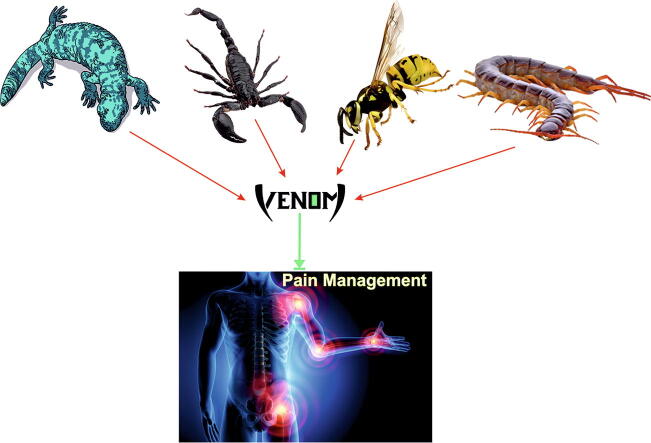

## Introduction

Several toxins have evolved among plants, animals, and microbes, as a part of their defence and to capture their prey (Lewis and Garcia, 2003). Many of these toxins are identified as peptides by nature. As many of such peptides are being established as highly selective and are relatively safer potent therapeutics (Pennington et al., 2018). The non-peptide toxins are typically orally active whereas the peptide toxins are found usually in animal venoms that are associated with special organs that are meant to deliver them *via* intramuscular, subcutaneous, or intravenous routes (Lewis and Garcia, 2003).

The venom of spiders, wasps, scorpions, and snakes is comprised of a mixture of bioactive proteins, peptides, and small molecules that can be proved deadly to the target prey (Yang et al., 2013). Among these, the venom peptides are one of the most aggressive molecules and have a lot of potentials that have been identified. The venom peptides by nature act as ligands of ion channels that are being extensively used in the pharmacological characterization of several ion channels and their receptors (Mobli et al., 2017). These are designed with disulfide bridges with a compact folding by nature that remains stable while injecting it in *in vivo* (Sabatier et al., 2013). In addition, they possess other favorable characteristic features such as small size with high stability; the diversity of the folds are used as an advantage for a functional derivation of the venom peptides (De Waard and Sabatier, 2006). The peptides after isolation as single compounds can be used at suitable concentrations, and thus venom-derived peptides are becoming as useful drugs (Pennington et al., 2018). As the overall number of these venom-derived bioactive peptides that are progressing successfully towards the treatment is still limited in the current therapeutic field, their prospects tend to be very promising (Pennington et al., 2018).

Snake venoms are advance killer machines, which are pharmacologically characterized natural toxins. They act on a myriad of exogenous targets such as ion channels, receptors and enzymes within cells or on the cell membrane. *Daboia russelii* (Russell’s viper) (Kameshwar et al., 2017); *Bungarus caeruleus* (Law et al., 2014); *Echis carinatus* (Katkar et al., 2014), and *Naja naja* (Neema et al., 2020) are the most perilous snakes in Indian subcontinent causing morbidity and mortality and also one among the big four family of venomous snake. The pathophysiology of *Daboia russelii* envenomation includes intense local effects (hemorrhage, edema, myonecrosis and alterations in coagulant system) and systemic effects (myotoxicity, neurotoxicity and systemic bleeding) (Nijaguna Prasad et al., 1996).

The toxins from the venoms can be either cytotoxins, cardiotoxin, hemotoxins, myotoxins, nephrotoxins, and neurotoxins. Snake venom sPLA_2_ from *Elapidae* and *Hydrophiidae* family belongs to the group I of sPLA_2_, whereas *Viperidae* and *Crotalidae* to group II, which is further subdivided into two main subgroups, depending on the residue at position 49 in the primary structure: D49 which are enzymatically active and K49 which possess low or no enzymatic activity (Lomonte and Calderón, 2003). Isoforms of sPLA_2_ which are predominately involved in inflammatory cascades of variety of tissue and cells are highly up regulated in response to inflammatory stimuli. Southern Indian regional *Daboia russelii* venom contains sPLA_2_s which are highly toxic and it constitute about 70% in the whole venom protein when compared to northern, western and eastern regions (Jayanthi and T. V. , 1988, Kasturi and T. V. G. , 1989).

Phospholipase A_2_ (PLA_2_) enzyme cleaves fatty acids at the *sn-2* position of glycerol backbone phospholipids releasing free fatty acid and lysophospholipid (Kameshwar et al., 2017, Dennis, 2000, Vivek et al., 2014). Isoforms of sPLA_2_ predominately involved in inflammatory cascade in variety of tissue and cells are highly up regulated in response to inflammatory stimuli both by external and internal is categorized as group as GIIa sPLA_2_ (Group-IIa sPLA_2_) enzyme. The reaction is of particular importance when the fatty acid released from the *sn-2* position is arachidonic acid. Arachidonic acid can be oxidatively metabolized by cyclooxygenase and lipooxygenase enzymes to prostaglandins, thromboxanes, prostacyclins and leukotrienes, which are the mediators of inflammation. Lysophospholipid containing a choline head group and an alkyl linkage in *sn-*1 position act as the proinflammatory mediator for platelet activating factor, these proinflammatory molecules enhances the severity of inflammation (Dennis, 2000, Burke and Dennis, 2009, Dileep and T. I., Sadasivan C. , 2011).

Pain is one of the common features experienced in most injuries and tissue damage. At present, the International Association for the Study of Pain (IASP) has defined pain as “An unpleasant sensory and emotional experience that is associated with actual or potential tissue damage or described in terms of such damage” (Raja et al., 2020). The venomous organisms have been frequently stereotyped as pain-inflicting and causing distresses which have been historically vilified by mankind. Surprisingly the same venoms that can cause pain if directly injected into a host animal can turn into next-generation analgesia when injected by a clinician (Trim and Trim, 2013). Pain is said to be the most common feature of any disease, that may accompany us from an early age with its protective mechanism while the body responds to any harmful stimulus (Swieboda et al., 2013). Pain is not only a direct output of nociception but also interacts with several inputs such as attention, affective dimensions, autonomic variables, immune variables, and more (Cortelli et al., 2013).

Based on their characteristic features, pain can be classified into acute pain, chronic pain, somatic pain, visceral pain, neuropathic pain, allodynia, hyperalgesia, and referred pain. Among these, chronic pain has been notoriously defined as the pain which can last much longer than its usual course of acute injury in any disease condition or the pain that may recur for months or years (Raffaeli and Arnaudo, 2017).

The general physiological process involved in mediating the pain is as follows (Cortelli et al., 2013):

**Table:** 

During any injury, an “inflammatory soup” is generally produced at the site of injury to stimulate and activate the nociceptors
↓
The afferent nociceptors from the periphery can transmit noxious signals to the projection neurons that are located in the dorsal horn of the spinal cord
↓
Cells in the dorsal horn are present as layers of physiologically distinct sections which are referred to as laminae. A subset of these projection neurons which are based on the type of synapse in the laminae formed by the nociceptive fibre can relay the information to the somatosensory cortex through the thalamus that can provide information about the spatial features and the intensity of the painful stimulus
↓
These projection neurons can engage the cingulate and insular cortices via connections with the parabrachial nucleus of the brainstem along with the amygdala is considered to be the ascending pathway that initiates the conscious perception of pain
↓
The ascending information can also prompt the neurons of the rostral ventral medulla and midbrain periaqueductal gray which can engage the descending feedback systems to regulate the output from the spinal cord
↓
Modulate pain sensation

The modulation event may take place at all the levels of nociceptive pathways via the primary afferent neuron, dorsal horn, and higher brain centers through up-regulation or down-regulation (Yam et al., 2018). The release of hormones and other substances such as endogenous opioids, GABA, glycine may have analgesic properties to limit pain sensation whereas, chemicals such as substance P (SP), glutamate, and aspartate can act on the spinal cord and excite the perception of pain (Cortelli et al., 2013).

The opioid receptors are localized in the peripheral and central nervous systems primarily within the pain pathways (Thobois et al., 2018). The most common μ receptors are present in the thalamus, striatum, locus coeruleus, while δ receptors can be located in the cortex, striatum, pons, and the κ ones may be found in the hypothalamus, nucleus accumbens, substantia nigra, ventral tegmental area, and solitaries tract nucleus (Thobois et al., 2018). Most of the pharmacologically useful proteins that can be represented as target classes for pain therapeutics are generally G-protein-coupled receptors (GPCRs), or enzymes, or ion channels and can be even growth factors (Trim and Trim, 2013). The GPCRs are commonly used as drug targets along with ion channel and enzyme inhibitors such as cycloxygenases (COX). A number of these venom peptide analgesics are being studied and have been shown to possess a potential role that can target specific classes of the pain mediating pathway.

## Methodology

This systematic review aimed to study the various venom in pain management. Several research articles are been published related to venom in pain management/ antinociception therapeutics in electronic form in international database of Scopus, Pubmed, Google Scholar and Science Direct from 2000 until 2021 were investigated. Search using Nociception, pain, venom, toxins, ion channel, analgesic, venomics, peptides, proteins, and FDA keywords with and/or operators in title and abstract was conducted. Studies results are reported in table with discussion.

### Bee venom-Melittin

Many studies have shown that melittin has potential glucose and lipid-lowering properties which mediates *via* several mechanistic pathways. The major anti-diabetes property of melittin is by increasing insulin secretion by depolarizing the pancreatic β-cells (Hossen et al., 2017). In another study by Duffy et al., reported that honey bee venom and melittin were found to suppress the activation of EGFR and HER2 by causing interference with their receptor phosphorylation in the plasma membrane studied in breast cancer cells. Further mutational studies found that the positive charge on the C-terminal sequence of melittin mediated the interaction with plasma membrane interaction and the anticancer property (Duffy et al., 2020) (Table 1). Melittin was able to reduce cell viability in leukemia cell lines such as acute lymphoblastic leukemia (CCRF-CEM) and chronic myelogenous leukemia (K-562) by inducing apoptosis *via* the intrinsic/mitochondrial pathway (Ceremuga et al., 2020). Mellitin was also shown to be fast-acting on cancer cell lines; was found to mediate cell membrane changes within one minute of exposure in AGS, COLO205, and HCT-15 cell lines (Soliman et al., 2019). *In vitro* studies have also shown melittin to exhibit antibacterial activity that was more pronounced against MRSA strains, in comparison with other Gram‑positive bacteria; further *In vivo* studies also showed MRSA‑infected mice treated with melittin, successfully exhibited recovery from MRSA‑infected skin wounds (Choi et al., 2015).

**Table 1:** 

**Sl no**	**Name of the molecule**	**Source**	**Sequence**	**Molecular Weight**	**Target**	**Status of the research**	**Drug approved/ designed**	**Marketing**	**Reference**
1	Melittin	*Apis mellifera*	GIGAVLKVLTTGLPALISWIKRKRQQ	2846.5 Da	Lymphatic System	Experimental Evidence At Protein Level	Approved	Prescription	https://pubchem.ncbi.nlm.nih.gov/compound/Melittin
2	Captopril	*Bothrops jararaca*	APPGlPSPPA	217.29 Da	Angiotensin-Converting Enzyme, 72 Kda Type Lv Collagenase	Completed	Approved	Prescription	https://pubchem.ncbi.nlm.nih.gov/compound/44093
3	Chlorotoxin	*Leiurus quinquestriatus*	MCMPCFTTDH QMARKCDDCC GGKGRGKCYG PQCLCR	3996 Da	Glioblastoma	Experimetal Evidence At Protein Level	Not approved	Research use only	https://pubchem.ncbi.nlm.nih.gov/compound/86278273
4	SLPTX Family	*Ethmostigmus rubripes*	MAFQVVLLSFALVVVLAVFD PCPSDCKCDVRSNQCRPVNDDVHPNVCINHYCIGVHLAKR EQRPELPHGA WDDSSEEKDS EASLA	111.148 Da	Histamine H1 Receptor, Histamine H2 Receptor	Experimetal Evidence At Protein Level	Not approved	Research use only	https://www.uniprot.org/uniprot/A0A023VZH1
5	Conotoxin M I	*Conus magus*	GRCCHPACGKNYSC	1437.6 Da	Voltage-Gated Sodium Channels	Experimetal Evidence At Transcript Level	investigational	Research use only	https://www.uniprot.org/uniprot/P01521
6	Exenatide	*Lacertilia*	HGEGTFTSDLSKQMEEEAVRLFIEWLKNGGPSSGAPPS	4187 Da	Glucagon-Like Peptide 1 Receptor	Experimental Evidence At Protein Level	Approved	Prescription	https://pubchem.ncbi.nlm.nih.gov/compound/45588096
7	Bivalirudin	*Hirudinea*	FPRPGGGGNGDFEEIPEEYL	2180.317 Da	Prothrombin	No Information Is Available On The Use Of Bivalirudin, An Alternative Drug Is Preferred	Approved	prescription	https://pubchem.ncbi.nlm.nih.gov/compound/16129704
8	Tirofiban	*Echis*	AGA	440.60 Da	Integrin Alpha-Iib, And Integrin Beta-3	Completed	Approved	Prescription	https://pubchem.ncbi.nlm.nih.gov/compound/60947
9	Apitoxin	*Apis mellifera*	GLGVLLVLTGLPALISTILALAGG	2803.4 Da	Central Nervous System	Completed	Approved	Prescription	https://pubchem.ncbi.nlm.nih.gov/compound/133082063
10	Cobrotoxin	*Naja Naja*	LECHNQQSSQTPTTTGCSGGETNCYKKRWRDHRGYRTERGCGCPSVKNGIEINCCTTDRCNN	6957 Da	Acetylcholine Receptor Inhibiting Toxin, Ion Channel Impairing Toxin, Neurptoxin, Postsynaptic Neurotoxin.	Experimental Evidence At Protein Level	Approved	Prescription	https://www.uniprot.org/uniprot/P60770
11	Desirudin	*Hirudinea*	VVYTDCTESGQNLCLCEGSNVCGQGNKCILGSDGEKNQCVTGEGTPKPQSHNDGDFEEIPEEYLQ	6963.52 Da	No Target Organ	Completed	Approved in some cases	It is used only for research and educational purposes.	https://pubchem.ncbi.nlm.nih.gov/compound/16129703
12	Enalapril	*Bothrops jararaca*	Unk-A-P-OH	376.453 Da	Angiotensin Converting-Enzymes	Completed	Approved	Prescription in some cases discontinued	https://pubchem.ncbi.nlm.nih.gov/compound/5388962
13	Eptifibatide	*Sistrurus miliarius*	CXGDWPC	832.0 Da	Integrin Beta-3	Ongoing	Approved	Prescription	https://pubchem.ncbi.nlm.nih.gov/compound/448812
14	Lixisenatide	*Heloderma suspectum*	HGEGTFTSDLSKQMEEEAVRLFIEWLKNGGPSSGAPPSKKKKK	4858.56 Da	Glucogon Like Peptide-1 Receptor	Completed	Approved	Prescription	https://pubchem.ncbi.nlm.nih.gov/compound/90472060
15	Ziconotide	*Conus magus*	CKGKGAKCSRLMYDCCTGSCRSGKC (synthetic)	2639.2 Da	Voltage-Dependent N-Type Calcium Subunit Alpha-1B, And Voltage-Dependent P/Q-Type Calcium Channel Subunit Alpha-1A	Withdrawn / Recruiting	Approved	Prescription	https://pubchem.ncbi.nlm.nih.gov/compound/16135415
16	Vespid chemotactic peptide T	*Vespa tropica*	FLPILGKILGGLL	1,354 Da	Inflammatory Cells	Ongoing	Approved	Prescription	https://www.uniprot.org/uniprot/P17231
17	Mastoparan	*Wasp venom*	INLKALAALAKKIL	1478.9 Da	Increases Gtpase Activity And Purify And Binds Gtp Binding Proteins	Experimental Evidence At Protein Level	investigation is in process	Research Use olnly	https://pubchem.ncbi.nlm.nih.gov/compound/6324633
18	Protonectin	*Protonectarina sylveirae*	ILGTILGLLK GL	1,211 Da	Defence Response To Bacterium	Experimental Evidence At Protein Level	Approved	Prescription	https://www.uniprot.org/uniprot/P0C1R1
19	Hainantoxin	*Haploplema hainanum*	MKASMFLALT GLALLFVVCY ASESEEKEFS NELLSSVLAV DDNSKGEERE CLGFGKGCNP SNDQCCKSSN LVCSRKHRWC KYEIGK	9,535 Da	Ion Channel Impairing Toxin, Neurotoxin, Pre-Synaptic Neurotoxin, Voltage Gated Sodium Channel Impairing Toxin	Experimental Evidence At Protein Level	Approved	Prescription	https://www.uniprot.org/uniprot/D2Y2D7
20	Batroxobin	*Bothrops atrox moojeni*	VIGGDECDIN EHPFLAFMYY SPRYFCGMTL INQEWVLTAA HCNRRFMRIH LGKHAGSVAN YDEVVRYPKE KFICPNKKKN VITDKDIMLI RLDRPVKNSE HIAPLSLPSN PPSVGSVCRI MGWGAITTSE DTYPDVPHCA NINLFNNTVC REAYNGLIPAK TLCAGVLQGG IDTCGGDSGG PLICNGQFQG ILSWGSDPCA EPRKPAFYTK VFDYLPWIQS IIAGNKTATCP.	28,189 Da	Defibrinogenating Agent	Not Yet Recruiting	Not approved	Not available	https://www.uniprot.org/uniprot/P04971
21	Apamin	*Apis mellifera*	CNCKAPEXALCARRCQQH	5,223 Dal	Central Nervous System, Ion channel	Not Yet Recruiting	Not Approved	Not available	https://pubchem.ncbi.nlm.nih.gov/compound/16129677
22	Arenicin-1	*Arenicola marina*	GFCWYVCYRNGVRVCYRRCN	22,497 Da	Antibiotics, Antimicrobial, Fungicide	Completed	Approved	Prescription	https://www.uniprot.org/uniprot/Q5SC60
23	Aurelin	*Aurelia aurita*	AACSDRAHGHICESFKSFCKDSGRNGVKLRANCKKTCGLC	4296.95 Da	Antibiotic, Antimicrobial	Completed	Approved	Prescription	https://pubmed.ncbi.nlm.nih.gov/16890198/
24	Hepcidin	*Oreochromis mossambicus*	DTHFPICIFCCGCCHRSKCGMCCKT	2789.4 Da	Antibiotics, Antimicrobial, Fungicides, Hormone	Completed	Approved	Prescription	https://pubchem.ncbi.nlm.nih.gov/compound/91864521
25	Scygonadin	*Scylla paramamosain*	MRSSLLLGLT VVVLLGVIVP PCMAGQALNK LMPKIVSAII YMVGQPNAGV TFLGHQCLVE STRQPDGFYT AKMSCASWTH DNPIVGEGRS RVELEALKGS ITNFVQTASN YKKFTIDEVE DWIASY	13,736 Da	Antimicrobial Activity Against Gram Positive Bacterium	Ongoing	Investigation is in process	Research use only	https://www.uniprot.org/uniprot/Q5D710
26	Hyastatin	*Hyas araneus*	MRVLLILVSL AALAHAESFL KSKTGYQGVQ TLPGFIGGSQ PHLGGGIGGG RPFISQPNLG GGIGSTRPFP RPQYGDYGSR NSCNRQCPST YGGRGICCRR WGSCCPTNYK G	13,452Da	C. Glutamicium, E.Coli, S. Aureus, P. Aeruginosa	Completed	Approved	Prescription	https://www.uniprot.org/uniprot/C4NZN9
27	Tauramamide	*Brevibacillus laterosporus*	YSLWR	864 Da	Multidrug Resistant Bacteria	Ongoing	investigation is in process	Research use only	https://pubchem.ncbi.nlm.nih.gov/compound/24180625
28	Centrocin 1b	*Strongylocentrotus droebachiensis*	MMIKVALVLC AIVATSMVCA KNFEEQDALD TLLNMMLSEE VASPDDAVAL QGWFKKTFHK VSHAVKSDIH AGQRGCSALG FSPEEARVKI LTAFPEMKEE DLTEEGVRAV CAGAHALGR	12,864 Da	Immune System	Ongoing	Not approved	Research use only	https://www.uniprot.org/uniprot/D8WN03
29	Calciseptine	*Dendroaspis polylepis*	RICYIHKASL PRATKTCVEN TCYKMFIRQTQ REYISERGCG CPTAMWPYQT ECCKGDRCNK	7044 Da	L-Type Calcium Channel Blocker	Ongoing	Not Approved	Research use only	https://www.uniprot.org/uniprot/P22947
30	μ-EPTX-Na1a	*Naja atra*	LKCHNTQLPFIYKTCPEGKNLCFKATLKKFPLKFPKRGCADNCPKNSALLKYVCCSTDKCN	7053.63 Da	Voltage-gated sodium channel Nav1.8	Ongoing	Not Approved	Research use only	https://pubmed.ncbi.nlm.nih.gov/30804211/

### Cobra venom-Captopril

In the early 1980′s the discovery of the ACE inhibitors and the isolation of captopril was one of the huge advancements in cardiovascular medicine, alongside the then used beta-blockers, calcium channel blockers, and statins (Péterfi et al., 2019). Captopril was the first ACE inhibitor approved for human use in 1981 which was developed based on the structure of bradykinin potentiating peptide that was isolated from the venom of the Brazilian pit viper, *Bothrops jararaca* (Péterfi et al., 2019). Captopril was designed as a miniature version of the original molecule due to its cost of isolation and inability to administer orally; with the addition of a succinyl group to a proline residue, thereby allowing its oral administration (Bordon, 2020). This amino acid residue which was found at the C-terminal of BPP5a is one of the most active peptides of bradykinin potentiating factor which was found to be responsible for interacting with ACE (Bordon, 2020) (Table 1). Captopril was added to standard therapy after acute myocardial infarction found that early or late administration improved survival and reduced cardiovascular morbidity, especially in selected high-risk patients (Plosker and McTavish, 1995).

### Scorpion venom-Chlorotoxin

Scorpion venom Chlorotoxin isolated from the Israeli scorpion *Leiurus quinquestriatus;* belongs to a family of chlorotoxin (CTX)-like peptides (Table 1) has been known for insecticidal activity, has potential ability to interact specifically with brain cell tumors such as gliomas (Arzamasov et al., 2014). It has been shown to block chloride channels which are expressed by the tumor glioma cells (Beeton, 2013). It was initially developed for the diagnosis and treatment of gliomas, recently it has been shown to specifically label cancer cells from solid tumors such as melanoma, small cell lung carcinoma, neuroblastoma, medulloblastoma, Ewing’s sarcoma, and pheochromocytoma (Sabatier and De Waard, 2013). In addition to chloride channels, other receptor cites such as matrix metalloproteinase-2 and Annexin-2A have also been claimed to be the binding sites for CTX (Dardevet et al., 2015).

### Centipede venom-Histamine

The centipede *Scolopendra viridicornis* venom has been shown to cause a local inflammatory response in individuals by inducing edema, leukocyte recruitment, and mast cells degranulation (Távora et al., 2016). None of the centipede venom peptides have yet progressed to late-stage preclinical studies or clinical trials (Undheim et al., 2016). Histamine can be used to produce a variety of effects within the body, including the contraction of smooth muscle tissues of the lungs, uterus, and stomach; the dilation of blood vessels, this increases permeability and lowers blood pressure; the stimulation of gastric acid secretion in the stomach; and the acceleration of heart rate (Table 1).

### Marine cone snail-Conotoxin

The venomous snail conotoxins are a valuable pharmacological probe and potential drug lead due to their highly specific nature and higher affinity towards ion channels, receptors, and transporters in the central nervous systems of target prey and humans (Gao et al., 2017). The conopeptide drug ω-conotoxin MVIIA isolated from *Conus magus*, has been clinically approved for the treatment of intractable pain, when directly administered to the spinal cord it specifically blocks a pain transmitting ion channel subtype called N-Type Ca^2+^ channels (Jin et al., 2019). Several conotoxin members have been identified with definitive pharmacological families that target the neuronal tissues: α (alpha), ι (iota), κ (kappa), and ρ (rho); which in turn target nicotinic acetylcholine receptors, voltage-gated Na channels, voltage-gated K channels, and α1-adrenoceptors, respectively (Sudewi et al., 2019). The other conopeptides are being evaluated for their potential antinociceptive properties and clot-dissolving cardioprotective agent (Becker and Terlau, 2008, Sousa et al., 2018).

More recently, KCP-400, which is also called as RgIA4 is derived from the venom of the *Conus regius*, a potent antagonist of the nicotinic acetylcholine receptor (nAChR). KCP-400 has been reported in its preclinical studies to provide relief against chronic pain (Pennington et al., 2018).

### Lizard venom-Exenatide

Exenatide is the synthetic form of a protein that mimics the action of glucagon-like peptide-1 (Table 1) which is found in the saliva of the Gila monster, shown to be important in glucose homeostasis and useful in the treatment of patients with diabetes mellitus (Triplitt and Chiquette, 2006). A synthetic analogue is known as Byetta also called exendin-4; exenatide was developed as a treatment for diabetes, shown to mimic GLP-1 by stimulating the GLP-1 receptor (Furman, 2012). Exendin‐4 enhanced the physiological functions of β‐cells and upregulated GLP‐1 receptors thereby reducing the plasma glucose levels. Exenatide has also been found to be useful in ameliorating neuropathy, nephropathy, and ventricular remodeling (Yap and Misuan, 2018).

### Leech venom-Bivalirudin

Bivalirudin is an analogue of hirudin is used in the treatment of deep vein thrombosis and repair of coronary angioplasty (Beeton, 2013). It is a bivalent direct thrombin inhibitor that binds to two distinct sites on thrombin- the active catalytic site and fibrinogen-binding site- exosite 1 (Warkentin, 2004). It was found to reduce the risk of bleeding and was approved by FDA for the treatment of unstable angina undergoing percutaneous transluminal coronary angioplasty (Bordon, 2020). As its binding to thrombin is reversible, Bivalirudin after the binding, is slowly cleaved by thrombin and hence thrombin activity gets transiently inhibited later its enzymatic activity is restored (Chudzinski-Tavassi et al., 2018).

### Saw-scaled viper snake-Tirofiban

Tirofiban is a non-peptide molecule that was the first antiplatelet drug derived from a snake venom protein is a low molecular weight reversible antagonist (Lazarovici et al., 2019). It has been shown to block the binding of fibrinogen to αIIbβ3 integrin (a fibrinogen receptor) and hence is used in the treatment of patients with unstable angina or NSTEMI who are undergoing PCI (Beeton, 2013, Lazarovici et al., 2019). Tirofiban thus reduces the risk of ischaemic complications in patients with unstable angina/non-Q-wave MI and also high-risk patients undergoing revascularisation when used against a background of heparin and aspirin, and hence can be used as an adjunct to heparin and aspirin in patients with acute coronary syndromes (McClellan and Goa, 1998).

### Honey bee venom-Apitoxin

The bee venom apitoxins from *Apis mellifera* have been shown to possess antitumor activities against different types of cancer cells such as breast, liver, blood, lung, skin, and prostate cancer cells (Wehbe et al., 2019). Apitoxin and its component melittin have been shown to possess potential application against oral pathogens with their antibacterial properties (Leandro et al., 2015). Apitoxin is also reviewed for its anti-inflammatory, anti-arthritic, and neuroprotective effects against Parkinson’s disease (PD), Alzheimer’s disease (AD), and amyotrophic lateral sclerosis (ALS) (Aufschnaiter et al., 2020). Importantly, Apitox® in a recent FDA phase 3 study has been implicated for the reduction of pain and increase in mobility in Osteoarthritis patients.

### Cobra venom-Cobrotoxin

Cobrotoxins are postsynaptic neurotoxins that bind to the acetylcholine receptors on the motor endplate (Warrell, 2020). Cobrotoxin has also been shown to be a potential drug candidate for chronic kidney diseases as its administration elevated anti-inflammatory cytokine IL-10 expression; inhibited phosphorylation of IκB-α and NF-κB p65 nuclear translocation; upregulated protein levels of podocyte-specific nephrin; downregulated the level of fibrosis-related TGF-β in Adriamycin- induced chronic nephropathy in a rat model (Wang et al., 2015). It has also been proposed to possess the potential to treat patients with COVID-19 or to inhibit SARS-COV-2 infection because it can inhibit the cytokine storm caused by SARS-COV2 infection; inhibit the proliferation of CD8 T-cells more than that of CD4 T cells to restore the CD4/CD8 ratio; inhibit lung inflammation, improve lung gas exchange function, and attenuating the development of fibrotic lesions in the lung; analgesics action providing relief to patients with muscle pain and headache; antiviral activity against SARS-COV2 (Lin et al., 2020).

### Leech venom-Desirudin

Desirudin is a recombinant analogue of hirudin which has promising anticoagulant potential approved by the FDA and is currently in use for the prevention of Deep Vein Thrombosis (DVT) following hip or knee replacement surgery (Abdualkader et al., 2011). Desirudin inhibits the different actions of thrombin such as fibrin formation, activation of coagulation factors V, VII, and XIII, and platelet aggregation, which results in a dose-dependent prolongation of a PTT (Koh and Kini, 2008). These are categorized under the class of Direct Thrombin Inhibitors (DTIs) which overcome the disadvantages of indirect thrombin inhibitors such as unfractionated heparins (Kong et al., 2014). It is also approved for treating heparin-induced thrombocytopenia (HIT) and for thrombotic prophylaxis following orthopedic surgery (Min et al., 2010).

### Jararaca pit viper snake venom-Enalapril

Enalapril is an angiotensin-converting enzyme (ACE) inhibitor that is used for the treatment of hypertension, congestive heart failure, post-myocardial infarction, and other indications. Enalapril is a pneumonia ACE inhibitor that reduces the risk of pneumonia by nearly one-third when compared to antagonists against calcium channel or beta-adrenoceptor in the treatment of hypertension (Davis et al., 2007). It was shown to be orally active, long-acting, with lesser side effects because of the absence of sulphydryl group; gets extensively hydrolyzed to its bioactive form *in vivo* to enalaprilat taking place in the liver (Gomez et al., 1985). In patients with congestive heart failure, administration of enalapril reduced mortality significantly by improving general signs & symptoms of left and right ventricular heart failure, reduction of heart size and blood pressure (Kjekshus and Swedberg, 1989).

### Pygmy rattle snake venom-Eptifibatide

Eptifibatide is a glycoprotein IIb/IIIa class platelet inhibiting drug used to reduce ischemic cardiac events approved by FDA for Acute Coronary Syndrome (ACS) and Percutaneous Coronary Intervention (PCI) (Phillips and Scarborough, 1997). A rupture in the atherosclerotic plaque or endothelial injury exposes the subendothelial matrix of the coronary blood vessel to the circulating platelets which in turn triggers a signaling cascade that leads to the activation of glycoprotein IIb/IIIa receptor (GpIIb/IIIa) (Sangkuhl et al., 2011). Eptifibatide belongs to the “disintegrin” peptide family which contains the sequence Arg-Gly-Asp (RGD) and mediates their effect by blocking αIIbβ3 integrin, which are the most powerful inhibitors of platelet aggregation (Lazarovici et al., 2019). It specifically functions by blocking the binding of adhesive proteins fibrinogen and von Willebrand factor to GP IIb/IIIa on the surface of activated platelets (Scarborough, 1999).

### Gila monster lizard venom-Lixisenatide

Lixisenatide is a 44 amino acid exendin-4-like analogue (Table 1) where It has C-terminal modification with the addition of 6 lysine residues and one proline deleted (Page, 2014). It is one of the anti-diabetic drugs which stimulate the GLP-1 receptor for binding of incretin-1 which in turn inhibits the release of glucagon, increases insulin secretion, delays gastric emptying, and is hence used for improving glycemic control in Type-2 diabetes mellitus (Brody, 2018). It also demonstrated mild improvements in HbA1c, with slightly lowered mean weight loss and better gastrointestinal tolerability with a lower incidence of hypoglycemia (Lear et al., 2019). Because of its higher CNS penetrating capability, it has also been implicated to have potential neuroprotective properties and the results have been promising at equivalent doses in *in vitro* models of neurodegeneration (Foltynie and Athauda, 2020). Unfortunately, its efficacy begins to reduce once anti-lixisenatide antibodies are produced in the body as it stimulates the immune system (Brody, 2018).

### Cone snail venom-Ziconotide

Ziconotide was the first marine-derived natural product approved by FDA in 2005 for its clinical application for neuropathic pain as a nonopioid analgesic (Shilpi and Uddin, 2020). It has been approved for the treatment of intractable cancer pain, phantom limb, chronic neuropathic pain, acute and chronic inflammatory pain and marketed as PRIALT® (Honore and Jarvis, 2007). Ziconotide targets presynaptic VGCCs (N-type voltage-gated calcium channels Ca_v_2.2) by binding to their α1B subunit obstructing the entry of Ca^2+^, which blocks neurotransmitter release and thereby prevents the synaptic transmission of pain sensation (Shilpi and Uddin, 2020). By blocking the neurotransmitter release from primary nociceptive afferents it prevents the transmission of pain signals to the brain (Marí and Tytgat, 2010). Ziconotide can also act on the Ca_v_3.1 and Ca_v_3.3 subtypes of the T-type calcium channel which are expressed extensively in the thalamus & cortex, are important for the regulation of thalamocortical signaling- an important component of sleep/wake regulation; has potential neuroprotective activity against epilepsy, schizophrenia, tremor or tinnitus (Barrow and Duffy, 2010).

### Vespa tropica venom chemotactic peptides [VCPs]

Chemotactic peptides are tridecapeptides in general with amphipathic, α-helical, linear, cationic, and C-terminal amide containing secondary structures with antimicrobial and hemolytic (Lee et al., 2016). In in vitro studies, these peptides displayed broad-spectrum antimicrobial activity against the standard and clinically isolated strains of bacteria and showed weak hemolytic activity towards human erythrocytes (Yang et al., 2013). The peptides displayed direct antimicrobial activity, enhanced the ability to attract leukocytes to the site of infection, and were also able to control inflammation in animal models (Silva et al., 2020). There were only three chemotactic peptides been reported which include Orancis-protonectin (OdVP2), EpVP6, and the one found in *R. brunneum* (named RbVP1 hereafter) (Lee et al., 2016). *In vitro* anti-tumor activities have also been studied in VCPs towards NIH:OVCAR-3 and SK-OV-3 ovarian cancer cell lines at concentrations higher than 10 µM (Abd El-Wahed et al., 2021).

### Wasp venom-Mastoparan

Mastoparan is a basic amphiphilic a-helical peptide that consists of 14 amino acid residues, hydrophobic and essential amino acids, and an amino acid C-terminus (Abd El-Wahed et al., 2021). They are generally polycationic, linear tetradecapeptide amides, rich in hydrophobic residues such as leucine, isoleucine, and alanine (Palma, 2013). Mastoparans are highly reactive against the cell membranes of bacteria, fungi, and erythrocytes, as well as mast cells, which results in antimicrobial, hemolytic, and Mast Cell Degranulation (MCD) activities (Lee et al., 2016). Its other biological effects include cytotoxic effects on tumor cells such as leukemia, myeloma, and breast cancer cells; induces mitochondrial permeability and powerful transition of mitochondrial permeability in homogeneous K562 cells (Abd El-Wahed et al., 2021). Mastoparan increases the GTPase activity and the rate of nucleotide-binding of several purified GTP-binding regulatory proteins (G proteins) which in turn couples cell-surface receptors to intracellular mediators; accelerated guanosine-5′-(3-O-thiotriphosphate) binding as a result of which G protein activation takes place in part by promoting the dissociation of bound GDP, (the mechanism by which receptors regulate G proteins) leading to cell toxicity (Higashijima et al., 1988). The specific effect of MCD depends on particular cell types such as the secretion of histamine if the cells are mast cells; serotonin if the cell type is platelets; catecholamines if the cell type are chromaffin cells; prolactin from the anterior pituitary, and even insulin if the cell type is pancreatic β-cells (Lee et al., 2016). Mastoparan B and Mastoparan M are other homologues isolated from other vespid venoms which vary concerning their antibacterial and anti-inflammatory properties (Abd El-Wahed et al., 2021).

### Brazillian wasp venom-Protonectin

Protonectin is a polyfunctional peptide that causes mast cell degranulation, releases lactate dehydrogenase (LDH) from mast cells, antibiotic activity against Gram-positive and Gram-negative bacteria, and chemotactic response for polymorphonucleated leukocytes (PMNL) (Baptista-Saidemberg et al., 2010). It has been shown potent antifungal activity and fungicidal activity against the candida fungi cells where Its action involved disrupting membrane integrity and inducing the production of cellular ROS (Wang et al., 2015). Its antibacterial activity is due to the formation of typical α-helical conformation in a membrane-mimicking environment, studied using molecular dynamics simulations (Wang et al., 2013). Protonectin also has a significant impact on lung cancer cells A549 and healthy lung fibroblast cells where it mediated the down-regulation of BMI-1 gene expression of cancer markers and up-regulation of the production of reactive oxygen species (ROS) (Eskandari et al., 2020).

### Oreochromis mossambicus venom-Ornithine

L-ornithine is a non-protein amino acid that is widely used to enhance human health as it has been reported to possess beneficial effects on the liver and the heart (Wu et al., 2020). Ornithine is formed mainly from L-glutamate in plants and synthesized from the urea cycle in animals as a result of the reaction catalyzed by enzymes in arginine (Seneca., 2007). L-Ornithine-L-Aspartate (LOLA) has been shown to promote hepatic ureagenesis and glutamine synthetase activity; also it promotes glutamine synthesis and possibly protein anabolism in skeletal muscle (Bajaj, 2012). Its ability to increase the buffering of ammonia during and after exercise is useful against skeletal muscle fatigue (Demura et al., 2010).

### Chinese bird spider-Hainantoxin

Hainantoxin-I is a novel peptide toxin (Table 1), isolated from the Chinese bird spider *Selenocosmia hainana* venom which has 33 amino acid residues with a disulfide linkage of I-IV, II-V & III-VI, assigned by partial reduction and sequence analysis (Li et al., 2004). The intermediate-conductance Ca^2+^-activated K^+^ (IK) channels (calcium/calmodulin-regulated voltage-independent K^+^ channels), whose activation (activation of IK currents) is important in blood vessels and respiratory tissues is mediated by hainantoxin-I (HNTX-I) as an IK-channel activator with little effect on voltage-gated Na^+^ and Ca^2+^ channels studied in rat dorsal root ganglion neurons and also the heterologous expression of voltage-gated rapidly activating delayed rectifier K^+^ channels in HEK293T cells (Huang et al., 2014). Hainantoxin-II (HnTx-II), is another neurotoxin isolated from the venom of the Chinese bird spider (*Haplopelma hainanum*) has higher insecticidal activity and lower lethiferous activity on mammals (Pan and Yu, 2010). Hainantoxin-III is a selective antagonist of neuronal tetrodotoxin-sensitive voltage-gated sodium channels; wherein it suppresses Nav1.7 current amplitude without altering any activation, inactivation, and repriming kinetics (Liu et al., 2013). Hainantoxin-IV (HNTX-IV) can specifically inhibit the neuronal tetrodotoxin-sensitive sodium channels and interact with neurotoxin receptor site 1 *via* a similar mechanism to that of TTX without affecting the activation and inactivation kinetics (Li et al., 2004). Hainantoxin-V is also a neurotoxic peptide that inhibits the tetrodotoxin-sensitive (TTX-S) sodium currents without any effects on tetrodotoxin-resistant (TTX-R) sodium currents on adult rats dorsal root ganglion neurons (Xiao and Liang, 2003).

### Tarantula venom peptides

*Theraphosa apophysis* venom has been recently reported to possess two tarantula-venom peptides (Tap1a and Tap2a). They have been identified to modulate the activity of both NaV and CaV3 channels. Inhibition of NaV and CaV3 channels by Tap1a and Tap2a has been identified as their mode of action in relieving from chronic visceral pain in a model of irritable bowel syndrome (Cardoso et al., 2021).

### Pit viper snake venom-Batroxobin

Batroxobin is a serine protease toxin (SVSP) isolated from the venom of many species of pit viper snakes. Batroxobin is an enzyme protein synthesized as a pre-proenzyme containing 18 residues pre-peptide with a six residue pro-peptide (Table 1) and has its clinical use as a defibrinogenating agent for various clinical conditions such as deep vein thrombosis, myocardial infarction, pulmonary embolus, central retinal vein occlusion, peripheral vascular disease, acute ischemic stroke, angina pectoris, glomerulonephritis, priapism, sickle cell crises and renal transplant rejection (Markland and Swenson, 2013). Batroxobin can boost the plasma plasmin concentration by inducing the release of plasminogen activator from the vascular endothelial cells and activate the fibrinolytic response which leads to the production of soluble non-functional fibrin degradation products and remove them from the plasma (Kaur et al., 2012). It effectively releases the fibrinopeptide A by cleaving the α‐chain of fibrinogen (Slagboom et al., 2017). It inhibits human neutrophil extracellular traps (NETs) induced by tumor necrosis factor-α (TNF-α) in the presence of human fibrinogen and protects against severe ischemic tissue injury and accelerates vascular and skeletal muscle regeneration (Masuda et al., 2019).

### Bee venom toxin-Apamin

Apamin is a neurotoxin peptide containing 18 amino acid residues which are tightly cross-linked by two disulfide bonds; mediates its pharmacological functions by irreversibly blocking Ca^2+^-activated K^+^ (SK) channels; also regulates gene expression of many signal transduction pathways involving cell development (Gu et al., 2020). Apamin specifically can block the Ca^2+^-activated potassium permeability which results from receptor activation (Strong and Brewster, 1992). As it is permeable to the blood–brain barrier, it can cause its effects on the CNS by different routes of administration; when peripherally applied it selectively and potently affects the K^+^ permeability of certain membranes such as smooth muscle of the gut (Palma, 2013). During this phase, there is a fall in the delayed hyperpolarization of cells, which can result in the elevated continuous firing of neurons in the mesencephalon and cerebellum which in turn elevates the cell sensitivity to excitatory inputs (Pucca et al., 2019). Apamin can activate the inhibitory muscarinic receptors of motor nerve terminals causing reduced neuromuscular transmission, which is undergoing experimental validation for potential treatment against diseases that present high muscle excitability, such as Parkinson's disease, learning deficit disorder, and other disabilities (Pucca et al., 2019).

### Polychaete: *Arenicola marina* venom-Arenicin-1

Arenicin-1 is a β-sheet antimicrobial peptide isolated from a marine polychaeta *Arenicola marina* a type of coelomocytes that has a potent, broad-spectrum antimicrobial activity (Orlov et al., 2019). It is a 21-residue peptide acting against pathogenic fungi by disrupting phospholipid membranes (Park and Lee, 2009). In studies done on *Candida* spp. there was an increase in the production of ROS and cytotoxic hydroxyl radicals production which lead to apoptosis due to mitochondrial dysfunction caused by arenicin-1 induced membrane depolarization and release of activated metacaspases (Cho and Lee, 2011). This further initiated apoptotic mechanism by causing plasma membrane depolarization; exposing the phosphatidylserine towards the outer surface; morphological changes in the nucleus and DNA structure (Cho and Lee, 2011). Based on these findings it is clear that arenicin-1 is a potent antifungal agent that primarily acts by inducing apoptosis. It also possesses antibacterial activity and displays hemolytic activity against human red blood cells (Lee et al., 2008).

## Jelly fish toxin-Aurelin

Aurelin is a novel antimicrobial peptide made up of 40 amino acids (Table 1) that has been shown to exhibit antibacterial activity against gram-positive and gram-negative bacteria (Ovchinnikova et al., 2006). It was purified from the mesoglea of a scaphoid jellyfish *Aurelia aurita* using preparative gel electrophoresis and RP-HPLC. It is initially synthesized as an 84-residue pre-pro-aurelin consist of a 22-residue putative signal peptide and a pro-stretch of 22 residues. It has no structural homology with any of the previously identified antimicrobial peptides but has partial similarity to defensins and K^+^ channel-blocking toxins of sea anemones belonging to ShKT domain family (Ovchinnikova et al., 2006). A recombinant peptide with (sup 15)N-labeled analogue was produced by overexpression in *Escherichia coli*, which was purified also had modest antibacterial properties and membrane activities (Shenkarev et al., 2012).

### *Oreochromis mossambicus* venom-Hepcidin

Hepcidins are important antimicrobial peptides that resist pathogenic infections consist of 88–91 amino acid residues which vary across the three different forms (Huang et al., 2007). They were isolated from tilapia (*Oreochromis mossambicus)*, and named TH1-5, TH2-2, and TH2-3 based on their sequence composition through hybridization of phage library; further synthetic peptides were also isolated and named as TH1-5 and TH2-3 which were also shown to have antimicrobial properties (Huang et al., 2007). Tilapia hepcidin (TH)1–5, has been also shown to induce an inflammatory response in HeLa cells indicating its potential application for cancer therapy (Chang et al., 2011). TH1-5 was found to be cytotoxic against MCF7 indicating it to be a promising cytotoxic peptide that warrants further studies as a potential anticancer agent for the treatment of breast cancer (Al-kassim Hassan et al., 2015). It was found to activate caspases-3/7 and − 9; suggesting induction of apoptosis via the intrinsic pathway providing further evidence for potential agent treatment breast cancer (Hassan et al., 2016).

### *Scylla serrata* venom-Scygonadin

Scygonadin comprises 102 amino acids (Table 1) with a theoretical molecular weight of 11.272 kDa having strong implications in providing reproductive immunity found generally in the seminal plasma of the mud crab, *Scylla serrata* (Li, 2013). It is antimicrobial offers protection against microorganisms due to its anionic group, pI 6.0, antibacterial activity against *A. hydrophila* (G-) and *M. leteus* (G + ) (Wang et al., 2006). Its 126 amino acids sequence constitutes a putative NH(2)-terminal signal sequence of 1–24 and a mature peptide 25–126 sequence (Wang et al., 2007). *P. pastoris*-derived recombinant scygonadin demonstrated a higher antimicrobial activity against pathogenic *Aeromonas hydrophila* showing salt-resistance and time-dependent killing kinetics; also antiviral potential demonstrating interference with replication of white spot syndrome virus (WSSV) in *in vitro*-cultured crayfish haematopoietic (Hpt) cells (Peng et al., 2012). The mature peptide expressed in *Escherichia coli* has an approximately 43 kDa fusion protein CKS-scygonadin was found to be highly stable, soluble, active against both Gram-positive and Gram-negative bacteria showing its antibacterial properties (Peng et al., 2010).

### *Hyas araneus* venom-Hyastatin

Hyastatin is an 11.7 kDa (Table 1) Gly-rich peptide isolated from the hemocytes of the spider crab *Hyas araneus* (Kang et al., 2015).It is made up of three distinct domains: N-terminal region rich in Glycine residues, short Proline or Arginine-rich region, and C-terminal region with six Cysteine residues resembling the one found in penaeidins; have shown antimicrobial properties against yeasts, and Gram-positive and Gram-negative bacteria (Sperstad et al., 2009). A recombinant product of Sp Hyastatin (from *Scylla paramamosain*) showed potent antimicrobial activity against *Staphylococcus aureus, Aeromonas hydrophila,* and *Pseudomonas fluorescens* with antimicrobial mechanism attributing to the ability to disrupt cell membrane integrity on Hyastatin treatment (Shan et al., 2016). As both native hyastatin and its N-terminus region can bind chitin, this may facilitate antifungal capability whereas its antibacterial properties can be attributed to the cysteine-rich C-terminus domain, since it is absent in the recombinant hyastatin which do not possess antibacterial properties (Smith et al., 2010).

### *Brevibacillus laterosporus* protein-Tauramamide

Tauramamide is a novel lipopeptide antibiotic produced by the culture of marine bacterial isolate *Brevibacillus laterosporus* PNG276 (Hassi et al., 2012). It is a linear lipopeptide that is made up of 7-methyloctanoic acid esterified on a pentapeptide chain (Yang et al., 2016). Tauramamide and its ethyl ester showed potent antibiotic activity against pathogenic *Enterococcus sp*. (Desjardine et al., 2007). it is made up of two D-amino acids with an acylated N-terminus (Debbab et al., 2010). Antimicrobial peptides, tauramamide, ethyl ester, thiopeptides, and depsipeptides, from marine bacterial origin showed effective inhibition of human pathogenic *Enterococcus sp* (Biswas et al., 2016).

### *Strongylocentrotus droebachiensis* venom-Centrocins

Venom from the green sea urchin *Strongylocentrotus droebachiensis*, centrocins 1 and 2 were purified from the coelomocyte extracts (Li et al., 2010). The centrocins possess an intramolecular heterodimeric structure with a heavy chain made up of 30 amino acids and a light chain made up of 12 amino acids (Björn et al., 2012). The full-length sequence of centrocin 1 is made up of 119 amino acids, whereas centrocin 2 is made up of 118 amino acids which both include a pre-pro-sequence consisting of 51 or 50 amino acids for centrocins 1 and 2, respectively, with 24 amino acids inter-chain between the heavy and light chain (Li et al., 2010). The nature of native peptides is cationic and showed potent activity against Gram-positive and Gram-negative bacteria. The synthesis and subsequent antimicrobial testing of individual monomers have shown that the cationic containing heavy chain and the original dimeric peptide are equally active which showed a broad spectrum antimicrobial property, resistance to physiological salt concentration, and anti-inflammatory properties (Björn et al., 2012). It was further demonstrated that centrocin 1 was produced by phagocytes, stored in granular vesicles which co-localizes with phagocytosed bacteria suggesting the formation of phagolysosomes for its microbicidal action (Li et al., 2010).

### Black mamba venom-Calciseptine

Calciseptine is the venom peptide of black mamba which is made up of 60-amino acids with four disulphide bonds (García et al., 2001). It is a smooth muscle relaxant and a cardiac contraction inhibitor with its physiological actions similar to that of 1;4 -dihydropyridines drugs which are important for cardiovascular disease treatment (de Weille et al., 1991). It selectively blocks the L-type Ca^2+^ channels and does not affect the N-type and T-type Ca^2+^ channels. It also acts as a channel agonist in skeletal muscle by modulating the permeation of divalent cations through L-type channels (García et al., 2001). It may bind to L-type Ca + channels, via the recognition site for 1, 4-dihydropyridines and hence do not affect the N-type or T-type Ca^2+^ channels (Harvey, 2013). The synthetic Calciseptine (CaS) has also shown inhibitory effects on the voltage-dependent Ca^2+^ current conductances of 25-pS and 12-pS channels in porcine tissue by reducing the mean open time and channel availability which resulted in decreased open probability of the 25-pS and 12-pS channels with different sensitivities (Teramoto et al., 1996).

Following are some noteworthy outcomes of venom peptide in pain management therapeutics.

• μ-EPTX-Na1a, a 62-residue three-finger peptide from the venom of the Chinese cobra (*Naja atra*), was shown to be a potent inhibitor of the voltage-gated sodium channel Nav1.8, that exhibits a high selectivity over other voltage-gated sodium channel subtypes and hence contributing to reducing inflammatory and neuropathic pain (Zhang et al., 2019).
• Crotalphine- a crotalid venom that is isolated and has been chemically characterized as a novel and potent antinociceptive peptide is responsible for the oral opioid activity that induced antinociception which was mediated via activation of kappa-opioid receptors (Konno et al., 2008).
• *Buthus martensii* (Karsch)- an analgesic-antitumor peptide (BmK AGAP) – is isolated from scorpion venom peptide that mediates analgesic properties by inhibiting the neuropathic and inflammation-associated pain through a MAPK-mediated mechanism (Ruan et al., 2018).
• PnPP-19- isolated from spider *Phoneutria nigriventer* was shown to induce central antinociception that involves the activation of CB1 cannabinoid, μ- and δ-opioid receptors (da Fonseca Pacheco et al., 2016).
• Apitox® in a recent FDA Phase 3 study has been implicated for the reduction of pain and increase in mobility in Osteoarthritis patients.
• KCP-400 (RgIA4) has been reported in its preclinical studies to provide relief against chronic pain relief (Pennington et al., 2018). Furthermore, research and drug development are in progress.

## Conclusion

Venoms are a rich source of novel compounds. Peptides of venoms of spiders, scorpions, cone snails, and especially snakes have been identified to physiologically play a role in protection and predation strategies of the organism. Their role in inhibiting the fast synaptic transmission have attracted researchers to choose them as plausible candidates in analgesic development. Venoms with exquisite potency and selectivity have been developed over millions of years of evolution, and we now have the means to maximise their potential.

Venom-based painkiller, which resembles the venom of cone snails, is commercially accessible. Sea anemone, spider, and scorpion venom proteins have also been discovered with potential biomedical anti-nociceptive use. Many notable advances have increased our understanding of venoms and its role in pain since the latter half of the twentieth century. The orientation of research in venom toxins to therapeutics has been changed phenomenally and more pain management strategies are being developed. Strategies and mechanisms established with the likes of developed therapies viz., Apitox®, PRIALT®, Crotalphine, KCP-400 can be extended with the advent of ‘-omics' tools and synthetic chemistry in this golden age of biological drug discovery, especially for pain research.

## CRediT authorship contribution statement

**V. Vidya:** Conceptualization, Writing – original draft. **Raghu Ram Achar:** Data curation, Conceptualization, Writing - review & editing. **M.U. Himathi:** Writing – original draft. **N. Akshita:** Writing - original draft. **T. Yogish Somayaji:** Writing - review & editing. **Vivek Hamse Kameshwar:** Conceptualization, Supervision, Writing - review & editing. **K. Byrappa:** Conceptualization, Data curation. **Dinesha Ramadas:** Writing – original draft.

## Declaration of Competing Interest

The authors declare that they have no known competing financial interests or personal relationships that could have appeared to influence the work reported in this paper.

## References

[b0005] Lewis R.J., Garcia M.L. (2003). Therapeutic potential of venom peptides. Nat. Rev. Drug Discovery.

[b0010] Pennington M.W., Czerwinski A., Norton R.S. (2018). Peptide therapeutics from venom: Current status and potential. Bioorg. Med. Chem..

[b0015] Yang S., Xiao Y., Kang D., Liu J., Li Y., Undheim E.A.B., Klint J.K., Rong M., Lai R., King G.F. (2013). Discovery of a selective NaV1.7 inhibitor from centipede venom with analgesic efficacy exceeding morphine in rodent pain models. Proc. Natl. Acad. Sci..

[b0020] Mobli M., Undheim E.A.B., Rash L.D. (2017). Advances in Pharmacology.

[b0025] Sabatier J.-M., De Waard M., Kastin A.J. (2013). Handbook of Biologically Active Peptides.

[b0030] De Waard M., Sabatier J.-M. (2006). Handbook of Biologically Active Peptides.

[b0035] Kameshwar V.H., Kumar J., Priya B.S., Swamy S.N. (2017). Synthesis, characterization and bioactivity studies of novel 1, 3, 4-oxadiazole small molecule that targets basic phospholipase A 2 from Vipera russelli. Mol. Cell. Biochem..

[b0040] Law A.D., Agrawal A.K., Bhalla A. (2014). Indian common krait envenomation presenting as coma and hypertension: A case report and literature review. J. Emerg. Trauma Shock.

[b0045] Katkar G.D., Sundaram M.S., Hemshekhar M., Sharma D.R., Santhosh M.S., Sunitha K., Rangappa K.S., Girish K.S., Kemparaju K. (2014). Melatonin alleviates Echis carinatus venom-induced toxicities by modulating inflammatory mediators and oxidative stress. J. Pineal Res..

[b0050] Neema K., Hamse Kameshwar V., Nafeesa Z., Kumar D., Babu Shubha P., Nagendra Prasad M., Swamy S.N. (2020). Serine protease from Indian Cobra venom: its anticoagulant property and effect on human fibrinogen. Toxin Reviews.

[b0055] B. Nijaguna Prasad, K. K., K. G. S. Bhatt, T. Veerabasappa Gowda. (1996) A platelet aggregation inhibitor Phospholipase A2, from russell’s Viper (Vipera russelli) venom: Isolation and characterization. Toxicon 34, 1173-1185. 10.1016/0041-0101(96)00033-5 8931258

[b0060] Lomonte B.A.Y, Calderón L. (2003). An overview of lysine-49 phospholipase A_2_ myotoxins from crotalid snake venoms and their structural determinants of myotoxic action. Toxicon.

[b0065] Jayanthi G.P., Gowda T.V. (1988). Geographical variation in India in the composition and lethal potency of Russell’s viper *(Vipera russelli)* venom. Toxicon.

[b0070] Kasturi S., Gowda T.V. (1989). Purification and characterization of a major Phospholipase A2 from Russell's viper *(Vipera russell)* venom. Toxicon.

[b0075] Dennis E.A. (2000). Phospholipase A2 in eicosanoid generation. Am. J. Respir. Crit. Care Med.

[b0080] Vivek H.K., Swamy S.G., Priya B.S., Sethi G., Rangappa K.S., Nanjunda S.S. (2014). A facile assay to monitor secretory phospholipase A2 using 8-anilino-1-naphthalenesulfonic acid. Anal. Biochem..

[b0085] Burke J.E., Dennis E.A. (2009). Phospholipase A2 structure/function, mechanism, and signaling. J Lipid Res.

[b0090] Dileep KV, T. I., Sadasivan C. (2011) Molecular Docking Studies of Curcumin Analogs with Phospholipase A2. Interdiscip Sci Comput Life Sci 3, 189-197. 10.1007/s12539-011-0090-9 21956741

[b0095] Raja S.N., Carr D.B., Cohen M., Finnerup N.B., Flor H., Gibson S., Keefe F.J., Mogil J.S., Ringkamp M., Sluka K.A., Song X.-J., Stevens B., Sullivan M.D., Tutelman P.R., Ushida T., Vader K. (2020). The revised International Association for the Study of Pain definition of pain: concepts, challenges, and compromises. Pain.

[b0100] Trim S.A., Trim C.M. (2013). Venom: the sharp end of pain therapeutics. Br. J. Pain.

[b0105] Swieboda P., Filip R., Prystupa A., Drozd M. (2013). Assessment of pain: types, mechanism and treatment. Ann. Agric Environ. Med. Spec..

[b0110] Cortelli P., Giannini G., Favoni V., Cevoli S., Pierangeli G. (2013). Nociception and autonomic nervous system. Neurological sciences : official journal of the Italian Neurological Society and of the Italian Society of Clinical Neurophysiology.

[b0115] Raffaeli W., Arnaudo E. (2017). Pain as a disease: an overview. J. Pain Res..

[b0120] Yam M., Loh Y., Tan C., Khadijah Adam S., Abdul Manan N., Basir R. (2018). General Pathways of Pain Sensation and the Major Neurotransmitters Involved in Pain Regulation. Int. J. Mol. Sci..

[b0125] Thobois, S., Brefel-Courbon, C., Le Bars, D., and Sgambato-Faure, V. (2018) Molecular Imaging of Opioid System in Idiopathic Parkinson's Disease, In International Review of Neurobiology pp 275-303, Elsevier. 10.1016/bs.irn.2018.07.029 30314599

[b0130] Hossen M.S., Gan S.H., Khalil M.I. (2017). Melittin, a Potential Natural Toxin of Crude Bee Venom: Probable Future Arsenal in the Treatment of Diabetes Mellitus. J. Chem..

[b0135] Duffy, C., Sorolla, A., Wang, E., Golden, E., Woodward, E., Davern, K., Ho, D., Johnstone, E., Pfleger, K., Redfern, A., Iyer, K. S., Baer, B., and Blancafort, P. (2020) Honeybee venom and melittin suppress growth factor receptor activation in HER2-enriched and triple-negative breast cancer. npj Precision Oncology 4. 10.1038/s41698-020-00129-0 PMC7463160 32923684

[b0140] Ceremuga M., Stela M., Janik E., Gorniak L., Synowiec E., Sliwinski T., Sitarek P., Saluk-Bijak J., Bijak M. (2020). Melittin—A Natural Peptide from Bee Venom Which Induces Apoptosis in Human Leukaemia Cells. Biomolecules.

[b0145] Soliman C., Eastwood S., Truong V.K., Ramsland P.A., Elbourne A. (2019). The membrane effects of melittin on gastric and colorectal cancer. PLoS ONE.

[b0150] Choi J.H., Jang A.Y., Lin S., Lim S., Kim D., Park K., Han S.-M., Yeo J.-H., Seo H.S. (2015). Melittin, a honeybee venom-derived antimicrobial peptide, may target methicillin-resistant Staphylococcus aureus. Mol. Med. Rep..

[b0155] Péterfi O., Boda F., Szabó Z., Ferencz E., Bába L. (2019). Hypotensive Snake Venom Components-A Mini-Review. Molecules (Basel, Switzerland).

[b0160] Bordon, K.D. C. F., Cologna, C. T., Fornari-Baldo, E. C., Pinheiro-Júnior, E. L., Cerni, F. A., Amorim, F. G., Anjolette, F. A. P., Cordeiro, F. A., Wiezel, G. A., Cardoso, I. A., Ferreira, I. G., Oliveira, I. S. d., Boldrini-França, J., Pucca, M. B., Baldo, M. A., and Arantes, E. C. (2020) From Animal Poisons and Venoms to Medicines: Achievements, Challenges and Perspectives in Drug Discovery. Frontiers in Pharmacology 11. 10.3389/fphar.2020.01132 PMC7396678 32848750

[b0165] Plosker G.L., McTavish D. (1995). Captopril. Drugs Aging.

[b0170] Arzamasov A.A., Vassilevski A.A., Grishin E.V. (2014). Chlorotoxin and related peptides: Short insect toxins from scorpion venom. Russ. J. Bioorg. Chem..

[b0175] Beeton C. (2013).

[b0180] Sabatier J.-M., De Waard M. (2013).

[b0185] Dardevet L., Rani D., Aziz T., Bazin I., Sabatier J.-M., Fadl M., Brambilla E., De Waard M. (2015). Chlorotoxin: A Helpful Natural Scorpion Peptide to Diagnose Glioma and Fight Tumor Invasion. Toxins.

[b0190] Távora B.C.L.F., Kimura L.F., Antoniazzi M.M., Chiariello T.M., Faquim-Mauro E.L., Barbaro K.C. (2016). Involvement of mast cells and histamine in edema induced in mice by Scolopendra viridicornis centipede venom. Toxicon.

[b0195] Undheim E.A.B., Jenner R.A., King G.F. (2016). Centipede venoms as a source of drug leads. Expert Opin. Drug Discov..

[b0200] Gao B., Peng C., Yang J., Yi Y., Zhang J., Shi Q. (2017). Cone snails: a big store of conotoxins for novel drug discovery. Toxins.

[b0205] Jin A.-H., Muttenthaler M., Dutertre S., Himaya S.W.A., Kaas Q., Craik D.J., Lewis R.J., Alewood P.F. (2019). Conotoxins: chemistry and biology. Chem. Rev..

[b0210] Sudewi A.A.R., Susilawathi N.M., Mahardika B.K., Mahendra A.N., Pharmawati M., Phuong M.A., Mahardika G.N. (2019). Selecting Potential Neuronal Drug Leads from Conotoxins of Various Venomous Marine Cone Snails in Bali, Indonesia. ACS Omega.

[b0215] Becker S., Terlau H. (2008). Toxins from cone snails: properties, applications and biotechnological production. Appl. Microbiol. Biotechnol..

[b0220] Sousa S.R., McArthur J.R., Brust A., Bhola R.F., Rosengren K.J., Ragnarsson L., Dutertre S., Alewood P.F., Christie M.J., Adams D.J., Vetter I., Lewis R.J. (2018). Novel analgesic ω-conotoxins from the vermivorous cone snail Conus moncuri provide new insights into the evolution of conopeptides. Sci. Rep..

[b0225] Triplitt C., Chiquette E. (2006). Exenatide: From the Gila Monster to the Pharmacy. J. Am. Pharm. Assoc..

[b0230] Furman B.L. (2012). The development of Byetta (exenatide) from the venom of the Gila monster as an anti-diabetic agent. Toxicon.

[b0235] Yap M.K.K., Misuan N. (2018). Exendin-4 from Heloderma suspectum venom: From discovery to its latest application as type II diabetes combatant. Basic Clin. Pharmacol. Toxicol..

[b0240] Warkentin T.E. (2004). Bivalent direct thrombin inhibitors: hirudin and bivalirudin. Best Pract. Res. Clin. Haematol..

[b0245] Chudzinski-Tavassi A.M., Faria F., Flores M.P.A. (2018).

[b0250] Lazarovici P., Marcinkiewicz C., Lelkes P.I. (2019). From Snake Venom’s Disintegrins and C-Type Lectins to Anti-Platelet Drugs. Toxins.

[b0255] McClellan K.J., Goa K.L. (1998). Tirofiban. *Drugs*.

[b0260] Wehbe R., Frangieh J., Rima M., El Obeid D., Sabatier J.-M., Fajloun Z. (2019). Bee Venom: Overview of Main Compounds and Bioactivities for Therapeutic Interests. Molecules.

[b0265] Leandro L.F., Mendes C.A., Casemiro L.A., Vinholis A.H.C., Cunha W.R., Almeida R., d., and Martins, C. H. G (2015). Antimicrobial activity of apitoxin, melittin and phospholipase A2 of honey bee (Apis mellifera) venom against oral pathogens. Anais da Academia Brasileira de Ciências.

[b0270] Aufschnaiter A., Kohler V., Khalifa S., Abd El-Wahed A., Du M., El-Seedi H., Büttner S. (2020). Apitoxin and Its Components against Cancer, Neurodegeneration and Rheumatoid Arthritis: Limitations and Possibilities. Toxins.

[b0275] Warrell D.A. (2020).

[b0280] Wang S.-Z., Xu Y.-L., Zhu Q., Kou J.-Q., Qin Z.-H. (2015). Cobrotoxin fromNaja naja atraVenom Ameliorates Adriamycin Nephropathy in Rats. J. Evid. Based Complementary Altern. Med..

[b0285] Lin F., Reid P.F., Qin Z.-H. (2020). Cobrotoxin could be an effective therapeutic for COVID-19. Acta Pharmacol. Sin..

[b0290] M. Abdualkader, A., Merzouk, A., Ghawi, A. M., Alaama, and Mohammed. (2011) Some Biological Activities of Malaysian Leech Saliva Extract. IIUM Engineering Journal 12.

[b0295] Koh C.Y., Kini R.M. (2008). Anticoagulants from hematophagous animals. Expert Rev. Hematol..

[b0300] Kong Y.I., Chen H.A.O., Wang Y.-Q., Meng L., Wei J.-F. (2014). Direct thrombin inhibitors: Patents 2002–2012 (Review). Mol. Med. Rep..

[b0305] Min G.-S., Sarkar I.N., Siddall M.E. (2010). Salivary Transcriptome of the North American Medicinal Leech, Macrobdella decora. J. Parasitol..

[b0310] Davis S., Enna S.J., Bylund D.B. (2007). xPharm: The Comprehensive Pharmacology Reference.

[b0315] Gomez H.J., Cirillo V.J., Irvin J.D. (1985). Enalapril. *Drugs*.

[b0320] Kjekshus J., Swedberg K. (1989). Enalapril for congestive heart failure. Am. J. Cardiol..

[b0325] Phillips D.R., Scarborough R.M. (1997). Clinical pharmacology of eptifibatide. Am. J. Cardiol..

[b0330] Sangkuhl K., Shuldiner A.R., Klein T.E., Altman R.B. (2011). Platelet aggregation pathway. Pharmacogenet. Genomics.

[b0335] Scarborough R.M. (1999). Development of eptifibatide. Am. Heart J..

[b0340] Page R.C.L. (2014).

[b0345] Brody T. (2018). *FDA's Drug Review Process and the Package Label* pp.

[b0350] Lear S., Amso Z., Shen W. (2019). Methods in Enzymology.

[b0355] Foltynie, T., and Athauda, D. (2020) Repurposing anti-diabetic drugs for the treatment of Parkinson's disease: Rationale and clinical experience, In Progress in Brain Research pp 493-523, Elsevier. 10.1016/bs.pbr.2019.10.008 32247373

[b0360] Shilpi J.A., Uddin S.J. (2020). Annual Reports in Medicinal Chemistry.

[b0365] Honore P., Jarvis M.F. (2007).

[b0370] Marí F., Tytgat J. (2010).

[b0375] Barrow J.C., Duffy J.L. (2010). Annual Reports in Medicinal Chemistry.

[b0380] Lee S., Baek J., Yoon K. (2016). Differential Properties of Venom Peptides and Proteins in Solitary vs. Social Hunting Wasps. *Toxins*.

[b0385] Yang X., Wang Y., Lee W.-H., Zhang Y. (2013). Antimicrobial peptides from the venom gland of the social wasp Vespa tropica. Toxicon.

[b0390] Silva O.N., Torres M.D.T., Cao J., Alves E.S.F., Rodrigues L.V., Resende J.M., Lião L.M., Porto W.F., Fensterseifer I.C.M., Lu T.K., Franco O.L., de la Fuente-Nunez C. (2020). Repurposing a peptide toxin from wasp venom into antiinfectives with dual antimicrobial and immunomodulatory properties. Proc. Natl. Acad. Sci..

[b0395] Abd El-Wahed A., Yosri N., Sakr H.H., Du M., Algethami A.F.M., Zhao C., Abdelazeem A.H., Tahir H.E., Masry S.H.D., Abdel-Daim M.M., Musharraf S.G., El-Garawani I., Kai G., Al Naggar Y., Khalifa S.A.M., El-Seedi H.R. (2021). Wasp Venom Biochemical Components and Their Potential in Biological Applications and Nanotechnological Interventions. Toxins.

[b0400] Palma, M.S. (2013) Hymenoptera Insect Peptides, In: Handbook of Biologically Active Peptides pp 416-422, Elsevier.

[b0405] Higashijima T., Uzu S., Nakajima T., Ross E.M. (1988). Mastoparan, a peptide toxin from wasp venom, mimics receptors by activating GTP-binding regulatory proteins (G proteins). J. Biol. Chem..

[b0410] Baptista-Saidemberg N.B., Saidemberg D.M., de Souza B.M., César-Tognoli L.M.M., Ferreira V.M.R., Mendes M.A., dos Santos Cabrera M.P., Neto J.R., Palma M.S. (2010). Protonectin (1–6): A novel chemotactic peptide from the venom of the social wasp Agelaia pallipes pallipes. Toxicon.

[b0415] Wang, K., Dang, W., Xie, J., Zhu, R., Sun, M., Jia, F., Zhao, Y., An, X., Qiu, S., Li, X., Ma, Z., Yan, W., and Wang, R. (2015) Antimicrobial peptide protonectin disturbs the membrane integrity and induces ROS production in yeast cells. Biochimica et Biophysica Acta (BBA) - Biomembranes 1848, 2365-2373. 10.1016/j.bbamem.2015.07.008 26209560

[b0420] Wang K., Dang W., Yan J., Chen R., Liu X., Yan W., Zhang B., Xie J., Zhang J., Wang R. (2013). Membrane Perturbation Action Mode and Structure-Activity Relationships of Protonectin, a Novel Antimicrobial Peptide from the Venom of the Neotropical Social Wasp Agelaia pallipes pallipes. Antimicrob. Agents Chemother..

[b0425] Eskandari R., Asoodeh A., Behnam-Rassouli F. (2020). The Therapeutic Effects of an Antimicrobial Peptide Protonectin (IL-12) on A549 Cancer Cell Line. Int. J. Pept. Res. Ther..

[b0430] Wu X.-Y., Guo X.-Y., Zhang B., Jiang Y., Ye B.-C. (2020). Frontiers in Bioengineering and Biotechnology 7.

[b0435] Seneca. (2007) Alkaloid Chemistry, In Alkaloids - Secrets of Life pp 61-139, Elsevier.

[b0440] Bajaj, J.S. (2012) Hepatic Encephalopathy, In Zakim and Boyer's Hepatology pp 267-282, Elsevier.

[b0445] Demura S., Yamada T., Yamaji S., Komatsu M., Morishita K. (2010). The effect of L-ornithine hydrochloride ingestion on performance during incremental exhaustive ergometer bicycle exercise and ammonia metabolism during and after exercise. Eur. J. Clin. Nutr..

[b0450] Li D., Xiao Y., Xu X., Xiong X., Lu S., Liu Z., Zhu Q., Wang M., Gu X., Liang S. (2004). Structure-Activity Relationships of Hainantoxin-IV and Structure Determination of Active and Inactive Sodium Channel Blockers. J. Biol. Chem..

[b0455] Huang P., Zhang Y., Chen X., Zhu L., Yin D., Zeng X., Liang S. (2014). The Activation Effect of Hainantoxin-I, a Peptide Toxin from the Chinese Spider, Ornithoctonus hainana, on Intermediate-Conductance Ca2+-Activated K+ Channels. Toxins.

[b0460] Pan J.Y., Yu Z.Q. (2010). Isolation and characterization of Hainantoxin-II, a new neurotoxic peptide from the Chinese bird spider (Haplopelma hainanum). Dongwuxue Yanjiu.

[b0465] Liu Z., Cai T., Zhu Q., Deng M., Li J., Zhou X., Zhang F., Li D., Li J., Liu Y., Hu W., Liang S. (2013). Structure and Function of Hainantoxin-III, a Selective Antagonist of Neuronal Tetrodotoxin-sensitive Voltage-gated Sodium Channels Isolated from the Chinese Bird Spider Ornithoctonus hainana. J. Biol. Chem..

[b0470] Xiao Y.-C., Liang S.-P. (2003). Purification and characterization of Hainantoxin-V, a tetrodotoxin-sensitive sodium channel inhibitor from the venom of the spider Selenocosmia hainana. Toxicon.

[b0475] Cardoso F.C., Castro J., Grundy L., Schober G., Garcia-Caraballo S., Zhao T., Herzig V., King G.F., Brierley S.M., Lewis R.J. (2021). A spider-venom peptide with multitarget activity on sodium and calcium channels alleviates chronic visceral pain in a model of irritable bowel syndrome. Pain.

[b0480] Markland, F.S., Swenson, S. (2013) Venombin A, In: Handbook of Proteolytic Enzymes pp 3025-3034, Elsevier.

[b0485] Kaur, P., Ghariwala, V., Yeo, K. S., Tan, H.Z., Tan, J.C.S., Armugam, A., Strong, P.N., and Jeyaseelan, K. (2012) Biochemistry of Envenomation, In: Advances in Clinical Chemistry pp 187-252, Elsevier. 10.1016/b978-0-12-394384-2.00007-3 22870591

[b0490] Slagboom J., Kool J., Harrison R.A., Casewell N.R. (2017). Haemotoxic snake venoms: their functional activity, impact on snakebite victims and pharmaceutical promise. Br. J. Haematol..

[b0495] Masuda H., Sato A., Shizuno T., Yokoyama K., Suzuki Y., Tokunaga M., Asahara T. (2019). Batroxobin accelerated tissue repair via neutrophil extracellular trap regulation and defibrinogenation in a murine ischemic hindlimb model. PLoS ONE.

[b0500] Gu H., Han S.M., Park K.-K. (2020). Therapeutic Effects of Apamin as a Bee Venom Component for Non-Neoplastic Disease. Toxins.

[b0505] Strong, P.N., Brewster, B.S. (1992) Apamin: A Probe for Small-Conductance, Calcium-Activated Potassium Channels, In Methods in Neurosciences pp 15-24, Elsevier.

[b0510] Pucca, M. B., Cerni, F. A., Oliveira, I. S., Jenkins, T. P., Argemí, L., Sørensen, C. V., Ahmadi, S., Barbosa, J. E., and Laustsen, A. H. (2019) Bee Updated: Current Knowledge on Bee Venom and Bee Envenoming Therapy. Frontiers in Immunology 10. 10.3389/fimmu.2019.02090 PMC6743376 31552038

[b0515] Orlov D.S., Shamova O.V., Eliseev I.E., Zharkova M.S., Chakchir O.B., Antcheva N., Zachariev S., Panteleev P.V., Kokryakov V.N., Ovchinnikova T.V., Tossi A. (2019). Redesigning Arenicin-1, an Antimicrobial Peptide from the Marine Polychaeta Arenicola marina, by Strand Rearrangement or Branching, Substitution of Specific Residues, and Backbone Linearization or Cyclization. Mar. Drugs.

[b0520] Park, C., Lee, D.G. (2009) Fungicidal effect of antimicrobial peptide arenicin-1. Biochimica et Biophysica Acta (BBA) - Biomembranes 1788, 1790-1796. 10.1016/j.bbamem.2009.06.008 19539603

[b0525] Cho, J., and Lee, D.G. (2011) The antimicrobial peptide arenicin-1 promotes generation of reactive oxygen species and induction of apoptosis. Biochimica et Biophysica Acta (BBA) - General Subjects 1810, 1246-1251. 10.1016/j.bbagen.2011.08.011 21875650

[b0530] Lee J.-U., Park K., Lee J.-Y., Kim J.-K., Shin S., Park Y., Hahm K., Kim Y. (2008). Cell Selectivity of Arenicin-1 and Its Derivative with Two Disulfide Bonds. Bulletin of The Korean Chemical Society.

[b0535] Ovchinnikova T.V., Balandin S.V., Aleshina G.M., Tagaev A.A., Leonova Y.F., Krasnodembsky E.D., Men’shenin A.V., Kokryakov V.N (2006). Aurelin, a novel antimicrobial peptide from jellyfish Aurelia aurita with structural features of defensins and channel-blocking toxins. Biochem. Biophys. Res. Commun..

[b0540] Shenkarev Z.O., Panteleev P.V., Balandin S.V., Gizatullina A.K., Altukhov D.A., Finkina E.I., Kokryakov V.N., Arseniev A.S., Ovchinnikova T.V. (2012). Recombinant expression and solution structure of antimicrobial peptide aurelin from jellyfish Aurelia aurita. Biochem. Biophys. Res. Commun..

[b0545] Huang P.-H., Chen J.-Y., Kuo C.-M. (2007). Three different hepcidins from tilapia, Oreochromis mossambicus: Analysis of their expressions and biological functions. Mol. Immunol..

[b0550] Chang W.-T., Pan C.-Y., Rajanbabu V., Cheng C.-W., Chen J.-Y. (2011). Tilapia (Oreochromis mossambicus) antimicrobial peptide, hepcidin 1–5, shows antitumor activity in cancer cells. Peptides.

[b0555] Al-kassim Hassan M., Azemin W.-A., Dharmaraj S., Mohd K.S. (2015). Cytotoxic effect of hepcidin (th1-5) on human breast cancer cell line (MCF7). Jurnal Teknologi.

[b0560] Hassan M., Azemin W.-A., Dharmaraj S., Mohd K. (2016). Hepcidin TH1-5 Induces Apoptosis and Activate Caspase-9 in MCF-7 Cells. J. Appl. Pharm. Sci..

[b0565] Li Y. (2013). Recombinant Production of Crab Antimicrobial Protein Scygonadin Expressed as Thioredoxin and SUMO Fusions in Escherichia coli. Appl. Biochem. Biotechnol..

[b0570] Wang K., Huang W., Yang M., Cai J. (2006). Scygonadin, a novel male-specific anionic antibacterial protein from Scylla serrata (forskål, 1775). *The FASEB Journal*.

[b0575] Wang K.-J., Huang W.-S., Yang M., Chen H.-Y., Bo J., Li S.-J., Wang G.-Z. (2007). A male-specific expression gene, encodes a novel anionic antimicrobial peptide, scygonadin, in Scylla serrata. Mol. Immunol..

[b0580] Peng H., Liu H.-P., Chen B., Hao H., Wang K.-J. (2012). Optimized production of scygonadin in Pichia pastoris and analysis of its antimicrobial and antiviral activities. Protein Expr. Purif..

[b0585] Peng H., Yang M., Huang W.-S., Ding J., Qu H.-D., Cai J.-J., Zhang N., Wang K.-J. (2010). Soluble expression and purification of a crab antimicrobial peptide scygonadin in different expression plasmids and analysis of its antimicrobial activity. Protein Expr. Purif..

[b0590] Kang H., Seo C., Park Y. (2015). Marine Peptides and Their Anti-Infective Activities. Mar. Drugs.

[b0595] Sperstad S.V., Haug T., Vasskog T., Stensvåg K. (2009). Hyastatin, a glycine-rich multi-domain antimicrobial peptide isolated from the spider crab (Hyas araneus) hemocytes. Mol. Immunol..

[b0600] Shan, Z., Zhu, K., Peng, H., Chen, B., Liu, J., Chen, F., Ma, X., Wang, S., Qiao, K., and Wang, K. (2016) The New Antimicrobial Peptide SpHyastatin from the Mud Crab Scylla paramamosain with Multiple Antimicrobial Mechanisms and High Effect on Bacterial Infection. Frontiers in Microbiology 7. 10.3389/fmicb.2016.01140 PMC4954822 27493644

[b0605] Smith V.J., Desbois A.P., Dyrynda E.A. (2010). Conventional and Unconventional Antimicrobials from Fish, Marine Invertebrates and Micro-algae. Mar. Drugs.

[b0610] Hassi M., El Guendouzi S., Haggoud A., David S., Ibnsouda S., Houari A., Iraqui M. (2012). Antimycobacterial activity of a Brevibacillus Laterosporus strain isolated from a moroccan soil. Brazilian Journal of Microbiology.

[b0615] Yang, X., Huang, E., Yuan, C., Zhang, L., Yousef, A. E., Dozois, C.M. (2016) Isolation and Structural Elucidation of Brevibacillin, an Antimicrobial Lipopeptide from Brevibacillus laterosporus That Combats Drug-Resistant Gram-Positive Bacteria. 82, 2763-2772. 10.1128/AEM.00315-16 PMC4836408 26921428

[b0620] Desjardine K., Pereira A., Wright H., Matainaho T., Kelly M., Andersen R.J. (2007). Tauramamide, a lipopeptide antibiotic produced in culture by brevibacillus laterosporus isolated from a marine habitat: structure elucidation and synthesis. J. Nat. Prod..

[b0625] Debbab A., Aly A.H., Lin W.H., Proksch P. (2010). Bioactive compounds from marine bacteria and fungi. Microb. Biotechnol..

[b0630] Biswas K., Paul D., Sinha S.N. (2016). Marine bacteria: a potential tool for antibacterial activity. Appl. Environ. Microbiol..

[b0635] Li C., Haug T., Moe M.K., Styrvold O.B., Stensvåg K. (2010). Centrocins: Isolation and characterization of novel dimeric antimicrobial peptides from the green sea urchin, Strongylocentrotus droebachiensis. Dev. Comp. Immunol..

[b0640] Björn C., Håkansson J., Myhrman E., Sjöstrand V., Haug T., Lindgren K., Blencke H.-M., Stensvåg K., Mahlapuu M. (2012). Anti-infectious and anti-inflammatory effects of peptide fragments sequentially derived from the antimicrobial peptide centrocin 1 isolated from the green sea urchin, Strongylocentrotus droebachiensis. *AMB Express*.

[b0645] García M.C., Hernández-Gallegos Z., Escamilla J., Sánchez J.A. (2001). Calciseptine, a Ca 2+ Channel Blocker, Has Agonist Actions on L-type Ca 2+ Currents of Frog and Mammalian Skeletal Muscle. J. Membr. Biol..

[b0650] de Weille J.R., Schweitz H., Maes P., Tartar A., Lazdunski M. (1991). Calciseptine, a peptide isolated from black mamba venom, is a specific blocker of the L-type calcium channel. Proc. Natl. Acad. Sci..

[b0655] Harvey A.L. (2013).

[b0660] Teramoto N., Ogata R., Kuriyama H., Okabe K., Kameyama A., Kameyama M., Watanabe T.X., Kitamura K. (1996). Effects of calciseptine on unitary barium channel currents in guinea-pig portal vein. Pflügers Arch. Eur. J. Physiol..

[b0665] Zhang F., Zhang C., Xu X., Zhang Y., Gong X., Yang Z., Zhang H., Tang D., Liang S., Liu Z. (2019). Naja atra venom peptide reduces pain by selectively blocking the voltage-gated sodium channel Nav1.8. J. Biol. Chem..

[b0670] Konno K., Picolo G., Gutierrez V.P., Brigatte P., Zambelli V.O., Camargo A.C.M., Cury Y. (2008). Crotalphine, a novel potent analgesic peptide from the venom of the South American rattlesnake Crotalus durissus terrificus. Peptides.

[b0675] Ruan, J.-P., Mao, Q.-H., Lu, W.-G., Cai, X.-T., Chen, J., Li, Q., Fu, Q., Yan, H.-J., Cao, J.-L., Cao, P. (2018) Inhibition of spinal MAPKs by scorpion venom peptide BmK AGAP produces a sensory-specific analgesic effect. Molecular Pain 14, 174480691876123. 10.1177/1744806918761238 PMC5844526 29424271

[b0680] da Fonseca Pacheco D., Freitas A.C.N., Pimenta A.M.C., Duarte I.D.G., de Lima M.E. (2016). A spider derived peptide, PnPP-19, induces central antinociception mediated by opioid and cannabinoid systems. J. Venomous Anim. Toxins Incl. Trop. Dis..

